# Non-pharmaceutical interventions and inoculation rate shape SARS-CoV-2 vaccination campaign success

**DOI:** 10.1017/S095026882100217X

**Published:** 2021-10-11

**Authors:** Marta Galanti, Sen Pei, Teresa K. Yamana, Frederick J. Angulo, Apostolos Charos, Farid Khan, Kimberly M. Shea, David L. Swerdlow, Jeffrey Shaman

**Affiliations:** 1 Department of Environmental Health Sciences, Mailman School of Public Health, Columbia University, 722 West 168th Street, New York, NY 10032, USA; 2 Medical Development and Scientific/Clinical Affairs, Pfizer Vaccines, Pfizer Inc., 500 Arcola Road, Collegeville, PA 19426, USA; 3 Patient and Health Impact, Pfizer Vaccines, Pfizer Inc., 500 Arcola Road, Collegeville, PA 19426, USA

**Keywords:** Covid-19, vaccination, NPIs

## Abstract

Nearly 1 year into the coronavirus disease 2019 pandemic, the first severe acute respiratory syndrome coronavirus 2 vaccines received emergency use authorisation and vaccination campaigns began. A number of factors can reduce the averted burden of cases and deaths due to vaccination. Here, we use a dynamic model, parametrised with Bayesian inference methods, to assess the effects of non-pharmaceutical interventions (NPIs) (such as social distancing, mask mandates, school and workplace closure), and vaccine administration and uptake rates on infections and deaths averted in the United States. We show that scenarios depicting higher compliance with NPIs avert more than 60% of infections and 70% of deaths during the period of vaccine administration, and that increasing the vaccination rate from 5 to 11 million people per week could increase the averted burden by more than one-third. These findings underscore the importance of maintaining NPIs and increasing vaccine administration rates.

## Introduction

The novel coronavirus severe acute respiratory syndrome coronavirus 2 (SARS-CoV-2), the causative agent of coronavirus disease 2019 (COVID-19), emerged in China during late 2019 and rapidly spread throughout the world. In March 2020, the World Health Organization (WHO) declared the COVID-19 a pandemic, and by January 2021 SARS-CoV-2 had caused more than 100 million confirmed COVID-19 cases and 2 million deaths worldwide [1]. A global effort to develop vaccines against SARS-CoV-2 began early in 2020, but for most of that year the only options for slowing transmission were non-pharmaceutical interventions (NPIs), including stay-at home orders, encouraging the use of face masks, limiting in-person work and school and social distancing.

In December 2020, the US FDA granted emergency use authorisation for two COVID-19 vaccines that demonstrated safety and high efficacy in phase 3 trials: Pfizer/BioNTech BNT162b2 and Moderna mRNA-1273 [2]. In light of limited supply, the Centers for Disease Control and prevention (CDC) recommended prioritising vaccination per the following phases: (1a) healthcare workers and long-term care facility residents, (1b) priority essential workers and persons ≥75 and (1c) other essential workers, persons 65–75 and adults with pre-existing conditions [3]. In the United States, administration of BNT162b2 began on 14 December 2020 and administration of mRNA-1273 began on 21 December 2020. BNT162b2 and mRNA-1273 use mRNA technologies and require two doses administered 3 and 4 weeks apart, respectively, to reach full ~95% efficacy [4, 5]. By the end of 2020, the United States has secured commitments for 400 million doses of these vaccines, which could be available for the US population by July 2021 [6, 7]. However, by 31 December 2020, fewer than 3 million doses had been administered, corresponding to 22.5% of the distributed doses at that time [8] and less than 15% of the anticipated target [9]. During January 2021, the rate of vaccine administration increased. Presently, BNT162b2 is authorised for adults ≥16 years of age and mRNA-1273 for adults ≥18 years, but additional trials are being conducted to assess safety, immunogenicity and efficacy in children and pregnant women [10, 11].

The present study uses a dynamical modelling approach to estimate the potential benefit of the vaccination campaign in the United States by evaluating the joint impact of vaccination and different effective levels of NPIs, which reduce the contacts between different age and population groups during vaccine rollout. Since the beginning of the pandemic, similar mathematical modelling approaches have been used at various geographical and temporal scale to estimate relevant epidemiology parameters for SARS-CoV-2 [12], to generate short-term forecast on cases, hospitalisation and deaths [13] and to evaluate the efficacy of different interventions within a range of potential scenarios in Europe, United States and Asia [14–20].

## Methods

**Fig. 1.**
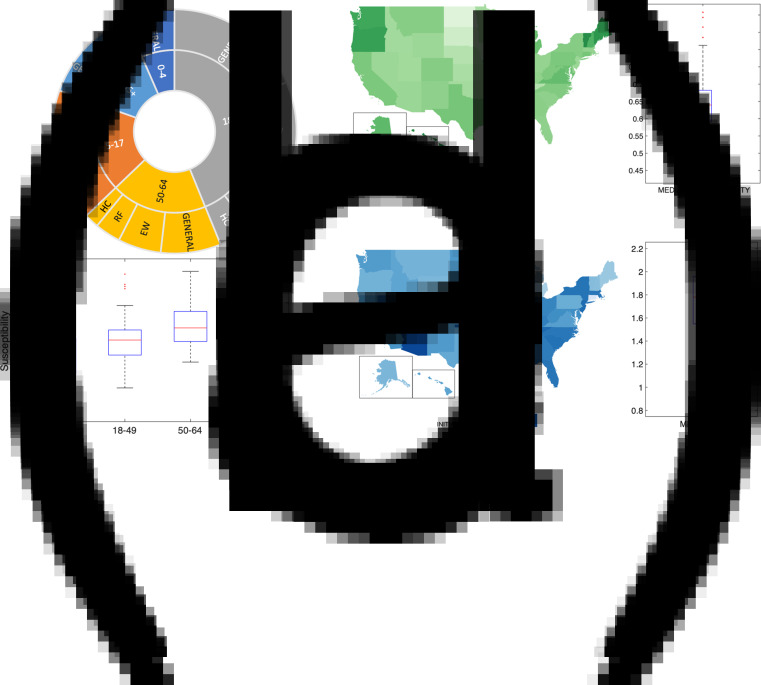
Initial conditions imposed on 10 January 2021. Panel (a) represents the structure of the population in 12 groups classified by combination of years of age (0–4, 5–17, 18–49, 50–64, ≥65), exposure status (HC, EW and general population) and health risk factor (RF and non-RF). See Supplementary Tables S1 and S2 for classification and overlapping factors. Panel (b) shows the initial susceptibility as a fraction of each state population. The boxplot shows the median, interquartile range and the full range of the distribution (outliers plotted in red) of the median values of population susceptibility for the 50 states and DC. Panel (c) shows the distribution of susceptibility for different age groups among states. Children 0–4 years and 5–17 years are combined as available from CDC seroprevalence data (see Supplementary Table S2 for details). Panel (d) presents a boxplot showing the median, interquartile range and full distribution range (outliers plotted in red) for the median values of the time-varying reproduction number *R_t_* on 10 January 2021 for the 50 states and DC.

In this analysis, we simulated and assessed the benefits of SARS-CoV-2 vaccination in the United States under varying levels of NPIs and differing vaccine administration and acceptance rates. Projections were made with a SEIRV (susceptible-exposed-infected-recovered-vaccinated) compartmental model run in isolation for all 50 states and the District of Columbia (DC), in which the population was stratified by age and priority group. Specifically, we stratified each state population by years of age (0–4, 5–17, 18–49, 50–64 and ≥65), adult exposure status (essential workers (EW), healthcare workers (HC) and other adults) and health risk status (presence or absence of one or more health risk factors for severe disease (RF)) (Fig. 1a, Supplementary Tables S1 and S2). The model was parametrised using posterior distributions estimated with a separate, non-stratified metapopulation model iterated through 10 January 2021 [21] and later adjusted for age and population types (see ‘Methods’ and Supplementary Table S2). Initial conditions and statistics for key epidemiological parameters are reported in Figure 1. The median estimated proportion of the state population susceptible (i.e. the population percentage not previously infected with SARS-CoV-2) on 10 January was 65%, and varied across states as shown in Figure 1b. Initial susceptibility for the vaccine scenario projections were varied based on seroprevalence differences in the population (Fig. 1c and Supplementary Table S2). The median national estimate of the time-varying reproduction number *R_t_* was 1.78 on 10 January; however, state-to-state heterogeneity of NPIs at that time is reflected in a broad distribution of *R_t_* values ranging from 0.8 to 2.2 (Fig. 1d). The estimate of the time-varying reproduction number on 10 January (median *R_t_* = 1.78) reflects reductions in opportunities for transmission due to NPIs, i.e. *R_t_* is a reduction of the basic reproduction number (*R*_0_). Relaxing (strengthening) the NPIs would increase (decrease) *R_t_* and, in turn, the theoretical threshold for herd immunity. Estimates of the basic reproduction number for SARS-CoV-2 in the United States vary across studies from 1.34 to 4 [22, 23]. In this paper, we present the results for *R*_0_ = 2.8; however, we also present results for *R*_0_ = 2.4 and *R*_0_ = 3.2 in the Supplementary material.

All vaccination scenarios assumed 400 million doses [6, 7] distributed to the US population according to ACIP prioritisation guidelines [3]. We considered phases 1a, 1b and 1c completed 10 days after (first) vaccination of a target coverage number of individuals. Once the prioritisation groups were vaccinated to target levels, vaccination was administered to other adults and children. The start date of the vaccination campaign was 14 December 2020, and, based on vaccination records [8], 5 million doses were administrated in the United States through the first 3 weeks of the vaccination campaign. Doses were allocated to the 50 states and DC in proportion to state population size, and two doses of vaccine were administered to all vaccinated individuals 3.5 weeks apart (see ‘Methods’ and Supplementary Tables S3 and S4 for details on vaccine modelling). Vaccination was administered regardless of prior history of infection and acted to prevent transmission to susceptible individuals. The impact of different scenarios was quantified in averted infections and deaths during the 15 months following 10 January. The (mean) averted burden of infection (both ascertained and unascertained) was measured for each intervention scenario *N_i_* with respect to the (mean) attack rate (AR) in reference scenario *N*_0_ (without vaccine and NPIs, see Supplementary Table S5) as:
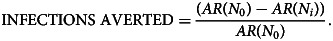


The same formula was used to quantify the averted deaths.

## Scenarios’ description

In this study, we assessed three effects: (1) the effect of NPIs under different vaccination scenarios, (2) the effect of vaccine administration rate under different NPIs mandates and (3) the effect of vaccine uptake under different NPI mandates. For the first analysis, we tested the effect of imposing different NPIs during the vaccination campaign. In this analysis, 5 million people received the first vaccine dose each week beginning week 4 of the campaign (Supplementary Table S4). We fixed the target coverage among different subpopulations at 80% for HC, 70% for risk groups (adults ≥65 and adults with RF) and 60% for other adults and children (up to available doses). In the main text, we present six different NPI scenarios (additional scenarios are described in Supplementary Table S5): *N*_0_ is the limit scenario without intervention (NPIs or vaccination); *N*_1_ has vaccination but NPIs are completely relaxed on 11 January 2021; *N*_2_ maintains NPIs at initial levels then completely relaxes them upon completion of phase 1a; *N*_3_ relaxes NPIs in three steps upon completion of phases 1a, 1b, 1c; *N*_4_ first strengthens NPIs then relaxes in three steps after completion of phases 1a, 1b, 1c and *N*_5_ maintains initial NPIs until (10 days after) 140 million people have initiated vaccination, then relaxes in three 1-month steps. In all scenarios, NPIs are implemented through a reduction of contact rates (see Supplementary Table S2 for details).

For the second analysis, we analysed how variations in the rate of vaccine administration impacted the cumulative infections and deaths averted due to vaccination. Specifically, we tested six vaccination schedules with 3, 5, 7, 9, 11 and 13 million people initiating vaccinations every week nationally. The six vaccination schedules were combined with four NPI policies: a ‘NO NPIs’ scenario with measures relaxed on 11 January 2021; a low distancing scenario (‘LOW’) with NPIs completely relaxed after one month; an intermediate distancing scenario (‘MED’) with NPI relaxation initiated after 1 month and gradually completed across 5 months and a strong distancing scenario (‘HIGH’) with measures first strengthened then gradually relaxed over 6 months. These scenarios correspond to scenarios *N*_1_, *N*_7_, *N*_8_ and *N*_9_ in Supplementary Table S5; all are characterised by a time-triggered relaxation of NPIs rather than a target-triggered relaxation in order to better isolate the effects of vaccination rate as phases 1a, 1b and 1c were reached at very different times across the six vaccination rates (e.g. the three prioritisation phases were completed after 254 days for 3 million vaccinated/week and after 62 days for 13 million/week). The target coverage in each group remained the same as in the previous analysis: 80% for HC, 70% for risk groups, 60% for other adults and children up to availability.

The third analysis examined the effects of vaccine uptake, specifically the percentage of each subpopulation able or willing to receive the vaccine (due to vaccine acceptance rates and difficulty accessing vaccination facilities), on population outcomes. Here, we assumed 5 million doses distributed per week beginning 11 January 2021. We assessed the effect of uptake by comparing the cumulative infections and deaths for the same four NPI scenarios, *N*_1_ (NO NPIs), *N*_7_ (LOW), *N*_8_ (MED), *N*_9_ (HIGH), considered in the previous analysis. Baseline coverage, *c*, remained 80% among HC, 70% among individual at risk and 60% among other adults and children up to availability. We then tested different uptake levels by increasing or decreasing the coverage of all groups by the same percentage (scenarios *c*_0.5_, *c*_0.75_, *c*_1.2_ are obtained multiplying the baseline uptake *c*, respectively, by 0.5, 0.75 and 1.2). Additionally, for scenario *c*_99_ target coverage was set to 99% for the whole population, and for scenario *c*_R_ target coverage was increased to 99% only for higher risk groups and kept at baseline for other groups.

## Results

### Effect of NPIs under fixed vaccination scenarios

**Fig. 2.**
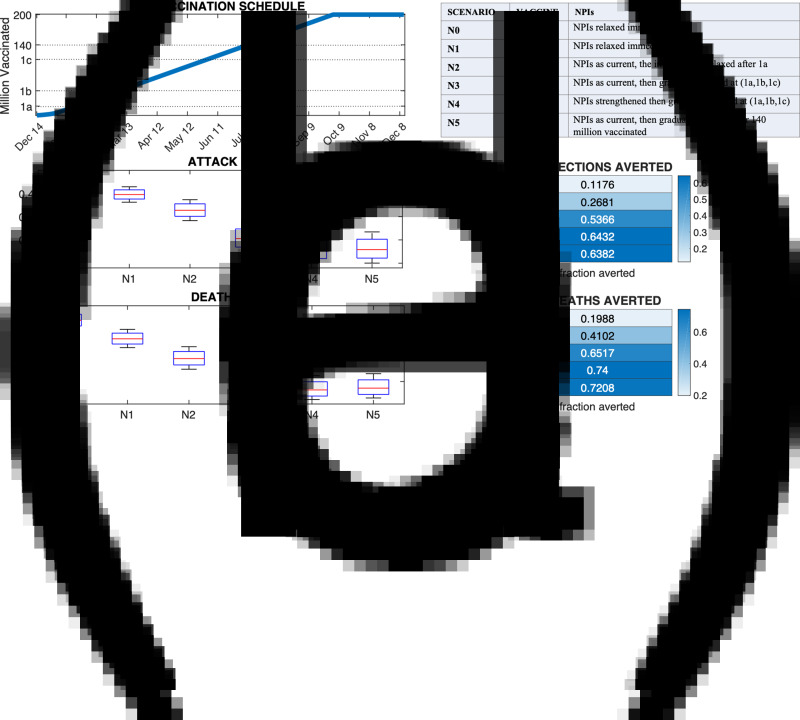
Effect of NPIs on infections and deaths with a fixed vaccination schedule. Panel (a) shows the vaccination schedule (first doses) (see also Supplementary Table S4). Phases 1a, 1b, 1c and 140 million vaccinated milestones are highlighted on the *y*-axis (the respective times on the *x*-axis do not include the additional 10 days required in the model for phase completion). The table panel summarises the six NPI scenarios. Note that NPIs are eventually completely relaxed in all scenarios. NPIs relaxation details are described in the ‘Methods’ section. Panels (b) and (c) show the attack rate and fractional reduction of infections for each scenario. Panels (2d) and (e) show the death rate and fractional reduction of deaths for each scenario. Note, the attack and death rate do not include infections and deaths prior to 11 January 2021.

On average, the three phases of vaccine prioritisation in [3] were completed, respectively, 23, 66 and 154 days after 11 January (timing differed in each state due to population structure) and 140 million vaccinated were reached 193 days after 11 January (Fig. 2a). Figure 2 compares the cumulative and averted burden of infection and death among the six different NPIs scenarios (described within ‘Methods’) characterised by different duration and strength of the NPIs imposed throughout the vaccination campaign. Other NPI scenarios, including scenarios in which relaxation was triggered by time and not phase completion are described in Supplementary Table S5 and the results are shown in Supplementary Figure S1.

**Fig. 3.**
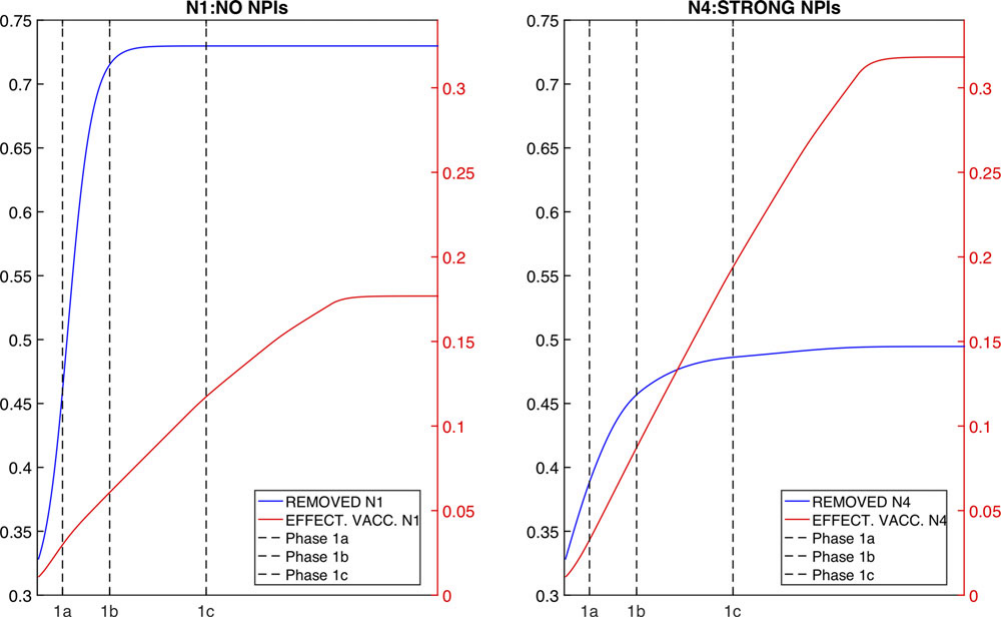
Effect of NPIs and vaccination on population immunity. Blue lines show the cumulative number of individuals no longer either susceptible or infected (i.e. recovered + deceased); red lines show the total effectively vaccinated (susceptible individuals who received the vaccine). The left panel shows the results from scenario *N*_1_; the right panel shows the results from scenario *N*_4_. Black vertical dashed lines mark the end of prioritisation phases.

In scenario *N*_0_ the cumulative attack rate in the overall population (for the period beginning 11 January 2021) was 44.8% and the cumulative death rate was 0.0015%. Adding vaccination (scenario *N*_1_) yielded an attack rate of 39.5% and death rate of 0.0012%, an 11.8% reduction of infections and a 19% reduction of deaths relative to scenario *N*_0_. Maintaining NPIs during the vaccination campaign allowed for much greater reductions: 20% to 60% of infections and 30% to 70% of deaths were averted depending on the strength of the NPIs maintained during the vaccination campaign. When NPIs were strengthened and gradually relaxed (*N*_4_) or maintained at initial levels for 6 months (scenarios *N*_5_ and *N*_6_, *N*_9_ in Supplementary Fig. S1) the attack rate in the population fell to roughly 15%. Figure 3 shows the faster spread of SARS-CoV-2 in the absence of NPIs: by the time phases 1a and 1b are completed in *N*_1_, 46% and 72% of the population is already immune (or deceased) by natural infection, whereas in *N*_4_ at the same time only 39% and 46% of the population have been infected (Fig. 3). These results were robust across a larger set of scenarios and for different estimates of *R*_0_ (Supplementary Text S2 and Fig. S1), and were consistent at the state level. Among the six NPIs scenarios described here, *N*_4_ and *N*_5_ had the lowest attack rate in all states (Supplementary Fig. S2). We also tested the sensitivity of the results to initial conditions (such as initial susceptibility) and vaccination setting (vaccine efficacy and the consequence of vaccination protecting against disease instead of infection) (Supplementary Text S3 and Figs S3–S6). Although estimates of infections and deaths depended strongly on some of these varied parameters, the general finding indicating the strong effect of NPIs held.

### Effect of vaccine deployment rate

**Fig. 4.**
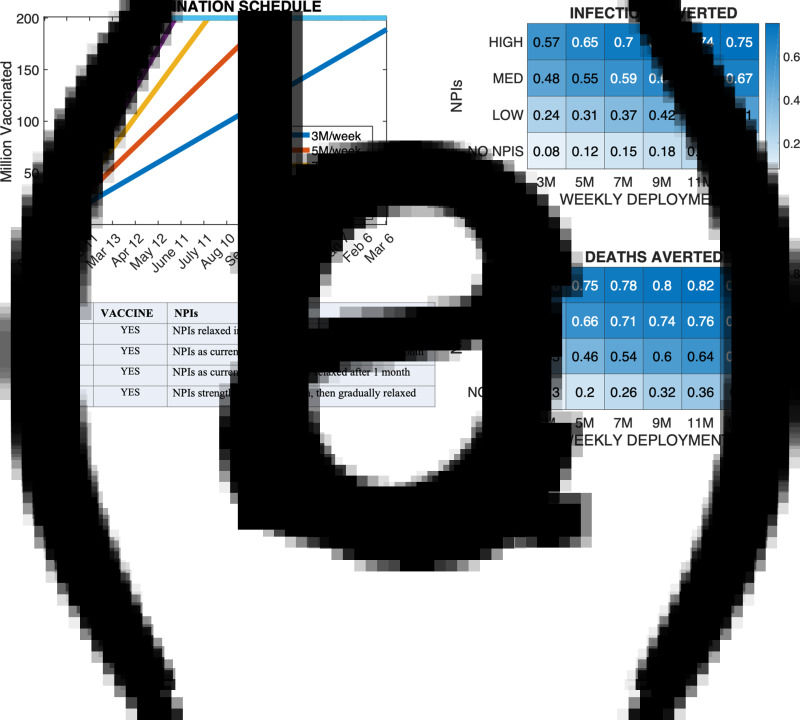
Effect of vaccine administration rate. Panel (a) shows the vaccine administration timeline for the six vaccine deployment rates simulated. Panels 3(b) and 3(c) show the fractional averted burden of infections and deaths for each combination of administration rate and NPI scenario relative to scenario *N*_0_. NPI scenarios NO NPIs, LOW, MED and HIGH correspond to scenarios *N*_1_, *N*_7_, *N*_8_ and *N*_9_ of Supplementary Table S5.

Expanding the number of doses administered per week from 5 to 7 million averted 3% to 6% more infections and 4% to 8% more deaths with respect to scenario *N*_0_; expanding from 5 to 11 million per week averted 9% to 16% more infections and 7% to 18% more deaths with respect to scenario *N*_0_ (Fig. 4). The effect of a faster deployment was stronger within scenarios characterised by weaker NPIs, because of the more rapid accumulation of infections in the first months. Results were robust to other estimates of *R*_0_ (Supplementary Text S4 and Fig. S7).

### Effect of vaccine uptake

**Fig. 5.**
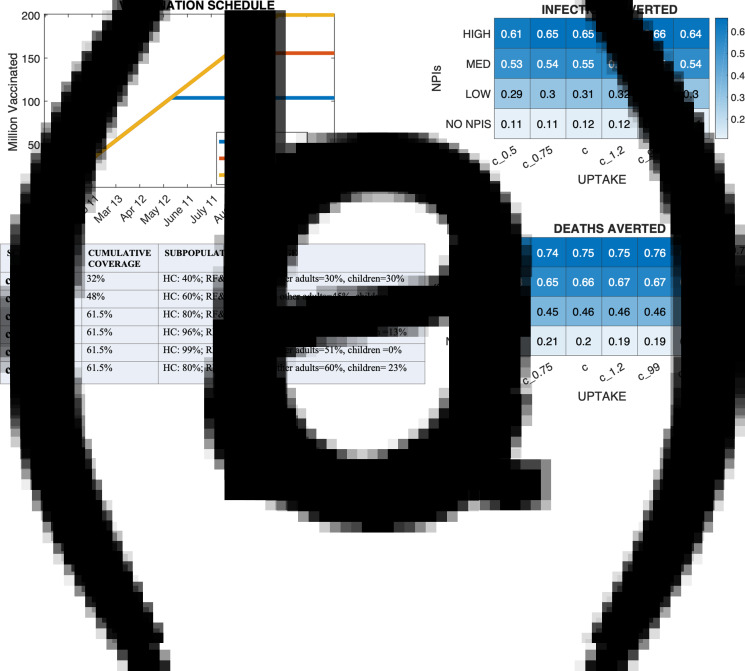
Effect of vaccine uptake on infections and deaths. Panel (a) shows the vaccine distribution timeline for the different vaccination uptake scenarios (*c*_0.5_, *c*_0.75_, *c*, *c*_1.2_, *c*_99_, *c*_R_). Panels b and c show the fractional averted burden of infections and deaths for each specific combination of vaccination uptake and NPI scenarios relative to baseline scenario *N*_0_. NPI scenarios NO NPIs, LOW, MED and HIGH correspond to scenarios *N*_1_, *N*_7_, *N*_8_ and *N*_9_ of Supplementary Table S5.

Given 400 million doses, the maximum percentage of the overall population that could be vaccinated was 61.5% overall, therefore scenarios *c*, *c*_1.2_, *c*_99_ and *c*_R_ (see ‘Methods’) reached the same cumulative coverage (see Fig. 5). The effect of a uniform increase or decrease in uptake across all groups was moderate, whereas a stronger impact on deaths averted was seen when increasing uptake solely for higher risk groups. Specifically, uniformly doubling uptake from 32% to 64% of the population averted 2% to 4% more infections and 3% to 5% more deaths with respect to the no intervention scenario *N*_0_ when some level of NPIs were also imposed. In the NO-NPI scenario (*N*_1_) doubling the uptake from *c*_0.5_ only averted 1% more infections and did not increase averted deaths. On the contrary, scenario *c*_R_ averted 4% more deaths with respect to *N*_0_ than the baseline scenario *c* with equal cumulative coverage (Fig. 5). In scenario *c*_0.5_ only 35% of the population at risk was vaccinated, but phases 1b and 1c started 2 weeks and 1 month, respectively, earlier than in scenario *c* (Supplementary Fig. S8). This earlier administration to phases 1b and 1c groups partially offset the lower vaccinated proportion. The averted infections for *c*_99_ varied minimally when increasing the total doses from 400 to 600 million (Supplementary Text S5). Results were robust to different estimates of *R*_0_ (Supplementary Fig. S9).

## Discussion

The recent advent of safe and efficacious SARS-CoV-2 vaccines could help end the pandemic. However, even in the most optimistic scenario, administering full vaccination with either BNT162b2 or mRNA-1273 to most of the population will take many months to complete, due to time required for production, distribution and administration of two doses. A number of factors could affect the rate of vaccine administration in the coming months: availability of doses, distribution of doses and management of the distributed stock by jurisdictions. According to the agreements stipulated in December 2020 between the US government and the vaccine manufacturers [6, 7], the United States has purchased enough doses of BNT162b2 and mRNA-1273 for full vaccination of more than 60% of the population. An additional 100 million doses of both BNT162b2 and mRNA-1273 have been recently contracted by the US government, other vaccine candidates are currently undergoing or completing phase 3 trials, and negotiation with manufacturers is ongoing [24]; thus, it is possible that additional vaccines and additional doses of BNT162b2 and mRNA-1273 may contribute to increased vaccine coverage in the coming months.

Here, we performed an analysis to test how the impact of the vaccination campaign (with a fixed total of 400 million doses) depends on three factors: (1) the NPIs imposed during vaccination, (2) the rate at which doses are administered and (3) the vaccine uptake within subpopulations differing by age, exposure status and health risk status. The strongest modulator of the impact of vaccination, measured by averted infections and deaths for a broad range of realistic scenarios was the enforcement of NPIs throughout the vaccination campaign. With stronger NPIs, virus transmission slows, allowing vaccination of more susceptible people prior to infection.

Overall, the vaccination campaign over the next several months has the potential to prevent infection of 20–40% of the US population; however, relaxing NPIs before attaining adequate vaccine coverage could result in infection of those individuals and further hospitalisations and mortality. In the scenario in which all NPIs were immediately relaxed 4 weeks into the vaccination campaign, the averted infections were only 14–27% of the number averted in the strongest NPIs scenarios, depending on the estimate of *R*_0_. When NPIs were maintained for a long time, hundreds of thousands of deaths were averted at the national level. Without NPIs, vaccination had a weaker impact because (1) herd immunity was approached earlier during the campaign because the susceptible pool was diminished due to a high attack and (2) the rate of *effective vaccination* (vaccination of susceptible individuals) was slower due to a lower susceptible fraction. Other modelling studies, carried out in the same time period and evaluating the impact of SARS-CoV-2 vaccination in conjunction with other public health measures, have also found that premature relaxation of NPIs could reduce vaccination benefits [14–20]. Our findings provide additional evidence for the importance of both maintaining NPIs during the vaccination campaign and a rapid vaccine rollout. However, we are now over one year into the pandemic; exhaustion and the economic toll of the pandemic cannot be discounted by policy makers in evaluating the extent and duration of the NPIs to be enforced during the next months.

The administration rate of the vaccine also had a strong impact in our analysis: increasing weekly vaccinations from 5 to 11 million, while keeping the cumulative availability fixed, reduced deaths by 17% to 20% with respect to the no intervention scenario across different estimates of *R*_0_. Increasing the speed of vaccine administration was particularly important for scenarios with reduced levels of NPIs. It is therefore essential to increase efforts to produce, distribute and administer the vaccine.

In the first weeks of the US vaccination campaign, only 20% of distributed doses were administered. By 28 January 2021, that percentage increased to about 50% [8]. Several factors need to be optimised: coordination between the federal government and individual states, management of the vaccine stocks by jurisdictions, operation of vaccination sites including coordination of personnel and strategies for facilitating population access and protocols to assure that vaccine doses are not wasted.

In our analysis, the effect of vaccine acceptance on the overall averted disease burden of the vaccine campaign was limited. Two processes appear to explain this result: (1) with a fixed administration rate of 5 million doses per month, the vaccination campaign had a greater impact in the first months when fewer natural infections had occurred, and when vaccine demand was greater than availability, even for low vaccination uptake scenarios; and (2) the prioritisation order did not place risk groups with higher mortality first in line for vaccination. As a result, increased coverage in not-at-risk groups had the effect of delaying administration to lower priority but more at-risk-groups, which, with the exception of LTCF residents, were included in phases 1b and 1c. In a low uptake scenario, the non-risk groups were processed faster, allowing the risk groups to be vaccinated earlier, albeit with a lower uptake. Increasing the uptake solely for the high-risk groups within the prescribed prioritisation order yielded the best outcome, consistent with other recent findings [25]. The averted infections in the scenario where acceptance was 99% in any population group varied minimally even when increasing the total doses from 400 to 600 million, indicating that an increase of the overall coverage is not particularly beneficial without an increase of the weekly vaccination rate.

This result, however, has to be evaluated within the limitations of our approach: first, we only considered mortality and infection, whereas the pandemic has an impact also on quality-adjusted life years and disability-adjusted life years, as well as occupational hazard and social disparity. Second, even though we characterised healthcare workers as having more work contacts than other adults, we didn't characterise those contacts as more likely to be with infected individuals. Therefore, the averted burden of infections with the current prioritisation could be underestimated.

Our analysis has several limitations. The high-dimensional model is sensitive to the choice of parameters and initial conditions. We tested finding sensitivity to several model parameters and even though the numbers of averted infections and deaths varied, sometimes largely, as with initial population susceptibility (see Supplementary Fig. S3) or the choice of *R*_0_, the overall conclusions of our analyses held.

In our study, we model vaccine as 95% efficacious (after two doses) in preventing infection and, as a consequence, transmission of SARS-CoV-2. However, the primary end points of the phase 3 trials for BNT162b2 and mRNA-1273 were efficacy against confirmed COVID-19 disease in vaccine recipients, not infection. There is some evidence [26] indicating vaccination reduces asymptomatic infection rates. However, at the moment observations are limited and more data are needed to distinguish vaccine efficacy in preventing infection and disease. Should the vaccines prove efficacious in preventing disease but not infection, the impact of vaccination on overall attack rate would likely be more limited than the effects shown here (see Supplementary Text S3 and Fig. S6); however, the importance of NPIs would be even more evident as transmission would be additionally supported by vaccinated individuals. In this instance, the immediate relaxation of NPIs during vaccine rollout would further counter vaccination benefits (Supplementary Text S3 and Fig. S6).

In this analysis, we also did not account for waning natural or vaccine-induced immunity or the emergence and dissemination of SARS-CoV-2 variants for which vaccines may be less efficacious [27] (however, we did test the sensitivity of results to lower efficacy of the vaccine (see Supplementary Text S3)). Evidence of re-infections with SARS-CoV-2 has been reported around the world [28–30]; however, more data are needed to understand the effect and time scale of these events at the population level. Should immunity prove to be short-lived, vaccination may need to be repeated every year or every few years for adequate coverage. A different model structure, accounting for loss of immunity, would be needed to quantify the burden of infection and deaths in this instance.

Overall, our findings indicate that vaccines can have a profound impact on the pandemic including prevention of many deaths. The public health objective is to vaccinate as many people as possible prior to infection. To do so, production, distribution and administration of vaccine must be accelerated and NPIs kept in place until enough doses are delivered to prevent sustained community transmission. Although the results presented here are outdated for the United States by the time of publication, i.e. 55% of the population had received at least one dose of vaccine by early July 2021, the findings are applicable to other countries and emphasise the importance of maintaining NPIs during vaccine campaigns. In Supplementary Text S6, we provide a comparison between the model projections and SARS-CoV-2 outcomes and interventions (NPIs and vaccination) observed in the first 6 months of 2021. NPI scenarios *N*_4_ and *N*_9_ best represented the estimated actual trajectory of *R*_t_ from January through June, although the fixed vaccination rate in the model did not reflect observed patterns of vaccination in the United States. The estimates of total infections for the *N*_4_ and *N*_9_ these scenarios span estimates of total infections during this first half of 2021.

## Data Availability

Inference data and model output are available on the *Github* repository: https://github.com/shaman-lab.

## Supplementary material

For supplementary material accompanying this paper visit http://dx.doi.org/10.1017/S095026882100217X. click here to view supplementary material
