# Supplementary material for: Non-pharmaceutical interventions and inoculation rate shape SARS-CoV-2 vaccination campaign success
Source: Epidemiol Infect. 2021 Oct 11;149:e222. doi: 10.1017/S095026882100217X (PMC8545880; doi:10.1017/S095026882100217X)
Supplement: Supplementary file 1 [file S095026882100217Xsup001.docx]

**SUPPLEMENTARY MATERIAL**

Non-pharmaceutical interventions and inoculation rate shape SARS-COV-2 vaccination campaign success

**List of Supplementary Materials**

**Supplementary Text S1-S6:**

**Supplementary Text S1: Methods**

**Supplementary Text S2: Extended model results on effect of NPIs.**

**Supplementary Text S3: Sensitivity Analysis**

**Supplementary Text S4: Extended model results for the effect of allocation rate**

**Supplementary Text S5: Extended model results on the effect of vaccine uptake**

**Supplementary Text S6: Comparison with observed outcomes and interventions**

**Supplementary Table S1-S5:**

**Supplementary Table S1**: **Population stratification**.

**Supplementary Table S2:** **Age- and population-specific parameter specifications**

**Supplementary Table S3**: **Vaccination parameters.**

**Supplementary Table S4**: **Weekly (first) vaccine distribution timeline (in millions).**

**Supplementary Table S5**: **All NPIs scenarios considered in the analysis.**

**Figure S1-S10:**

**Supplementary Figure S1 Attack rate and death rate for all NPIs**

**Supplementary Figure S2 Attack rate by state in scenarios N0 to N5**

**Supplementary Figure S3**: **Sensitivity to variation of initial susceptibility**

**Supplementary Figure S4:** **Attack rate and death rate for vaccine efficacy reduced by 20%**

**Supplementary Figure S5: Sensitivity of the attack rate to varying the relative ratios of contact rates**

**Supplementary Figure S6: Comparison between attack rate and death rate for disease-blocking vaccination and infection-blocking vaccination**

**Supplementary Figure S7:** **Averted burden for varying administration rates.**

**Supplementary Figure S8: Effect of NPIs and vaccination uptake on population immunity**.

**Supplementary Figure S9: Averted burden for varying uptake scenarios.**

**Supplementary Figure S10: model-generated posterior national estimates of R_t_ from January 10 through July 4, 2021.**

**Supplementary References 31-46.**

**Supplementary Text S1: Supplementary Methods**

**1.1 Model Description**

The principal model is a single location Susceptible-Exposed-Infected-Recovered-Vaccinated (SEIRV) compartmental structure run in isolation for each state in the US. We accounted for 5 age groups and 4 population types for the adult categories: healthcare workers (HC), essential workers (EW), individuals with pre-existing health conditions known to be risk factors for severe disease (RF) and persons other than HC, EW and RF (general) (see Table S1). We assume the 12 groups are mutually exclusive. The compartmental model is a modification of the structure that our group has used for inference and forecast for multiple infectious diseases, including influenza (*31*).

**Table S1**: **Population stratification**

|  | **AGE GROUPS** | **POPULATION-TYPE** |
| --- | --- | --- |
| 1 | 0-4 | general |
| 2 | 5-17 | general |
| 3 | 18-49 | general |
| 4 | 18-49 | HC |
| 5 | 18-49 | EW |
| 6 | 18-49 | RF |
| 7 | 50-64 | general |
| 8 | 50-64 | HC |
| 9 | 50-64 | EW |
| 10 | 50-64 | RF |
| 11 | > 65 | general |
| 12 | > 65 | RF |

Broadly, the model distinguishes between reported infections *I* and unreported infections *U*, with the former being more contagious and likely transmit virus. Natural infection (both reported and unreported) is assumed to confer permanent immunity. Vaccines are administered according to a specific prioritization calendar, and individuals are vaccinated regardless of previous infection record. However, only susceptible individuals receiving the vaccine transition to the Vaccinated compartment. The vaccine is administered in two doses 3.5 weeks apart to all recipients. Vaccine efficacy is modeled as a 3-step function with values:

$\epsilon_{1}$ until 12 days after first dose,

$\epsilon_{2}$12 days after first dose to 1 week after second dose

$\epsilon_{3}$ after 1 week from second dose.

Estimates of vaccine efficacy and further details on vaccination are given in section 1.2.3 below and Supplementary Table S3.

The complete stratified model for each of the 50 states and DC, s=1:51, is:

$$\forall i =1:12 \left\{ \begin{aligned} \frac{dS_{i}}{dt}=-\beta_{0}S_{i}\sum_{j=1}^{12} c_{i,j}\frac{{(I}_{j}+rU_{j})}{N_{j}}-\nu_{i}S_{i}-\varphi\\ \frac{d{V1}_{i}}{dt}=\nu_{i}S_{i}-{V1}_{i}\frac{1}{P_{1}}-\beta_{0}\left( \epsilon_{1}{V1}_{i} \right)\sum_{j=1}^{12} c_{i,j}\frac{{(I}_{j}+rU_{j})}{N_{j}} \\ \frac{d{V2}_{i}}{dt}={V1}_{i}\frac{1}{P_{1}}-{V2}_{i}\frac{1}{P_{2}}-\beta_{0}\left( \epsilon_{2}{V2}_{i} \right)\sum_{j=1}^{12} c_{i,j}\frac{{(I}_{j}+rU_{j})}{N_{j}} \\ \frac{d{V3}_{i}}{dt}={V2}_{i}\frac{1}{P_{2}}-\beta_{0}\left( \epsilon_{3}{V3}_{i} \right)\sum_{j=1}^{12} c_{i,j}\frac{{(I}_{j}+rU_{j})}{N_{j}} \\ \frac{dE_{i}}{dt}=\beta_{0}S_{i}\sum_{j=1}^{12} c_{i,j}\frac{{(I}_{j}+rU_{j})}{N_{j}}-E_{i} \frac{1}{Z} \\ \frac{d\tilde{E}_{i}}{dt}=\beta_{0}\left( \epsilon_{1}{V1}_{i}+\epsilon_{2}{V2}_{i}+\epsilon_{3}{V3}_{i} \right)\sum_{j=1}^{12} c_{i,j}\frac{{(I}_{j}+rU_{j})}{N_{j}}-\tilde{E}_{i} \frac{1}{Z} \\ \frac{dI_{i}}{dt}=\alpha_{i} E_{i} \frac{1}{Z}+\tilde{\alpha}_{i} \tilde{E}_{i} \frac{1}{Z}-\frac{1}{D} I_{i}+\varphi\alpha_{i} \\ \frac{dU_{i}}{dt}=\left( 1-\alpha_{i} \right)E_{i} \frac{1}{Z}+\left( 1-\tilde{\alpha_{i}} \right)\tilde{E}_{i} \frac{1}{Z}-\frac{1}{D} U_{i}+\varphi(1-\alpha_{i}) \\ N_{i}=S_{i}+E_{i}+\tilde{E}_{i}+I_{i}+U_{i}+{V1}_{i}+{V2}_{i}+{V3}_{i}+R_{i}. \end{aligned} \right.$$

(1)

For each subpopulation *i,* *S_i_, E_i_,* $\tilde{E}$*_i_, I_i_, U_i_, V1_i,_ V2_i,_ V3_i,_* and *R_i_* represent the susceptible, exposed, exposed vaccinated, infected reported, infected unreported, vaccinated (in the 3 phases) and recovered populations, $D$ the duration of infectious period, Z the latency period, and *N* the population size. We distinguish between Exposed and Exposed Vaccinated to allow for a different probability of developing reported infection: $\alpha_{i}$ and $\tilde{\alpha}_{i}$the ascertainment rate of infection, respectively, for unvaccinated and vaccinated individuals. Other parameters are: $\varphi$the travel-related importation of SARS-COV-2 into the model domain, $\nu_{i}$ the vaccination rate, and *r* the decreased probability of transmission for unreported infectious individuals. We allow the transmission rate to vary through specific age-dependent contact rates $c_{i,j}$ between individuals in age groups *N_i_* and *N_j_*, such that the full transmission term is ${(\beta}_{0}S_{i}+{\epsilon_{1}\beta}_{0}{V1}_{i}+{\epsilon_{2}\beta}_{0}{V2}_{i}+{\epsilon_{3}\beta}_{0}{V3}_{i})\sum_{j=1}^{12} c_{i,j}\frac{{(I}_{j}+rU_{j})}{N_{j}}$. In the present model, we do not consider waning immunity and the possibility of reinfection. Deaths were calculated from model output using a Case Fatality Rate (CFR) specified by age and pre-existing condition based on case report CDC data (see Supplementary Table 2 for details).

One hundred ensemble projections were simulated in each state for 450 days starting from initial conditions estimated on January 10, 2021. Projections for each state were initialized using posterior estimates derived from a separate metapopulation model-Bayesian inference system, described in Section 1.2.1.

**1.2 Initialization of parameters**

Our strategy for defining the distributions of parameters and variables in system (1) is based on the following steps:

1. We estimated the *population-level* distribution (interquartile range) of the epidemiological parameters on January 10, 2021 in each State with a non-stratified metapopulation model-Bayesian inference framework.
2. We combine the *population-level* estimates with information on population structure and age-specific infection and seroprevalence rates to stratify the initial conditions by age and population type (see Supplementary Table S2 for details on specific parameters and variables).
3. We initialized system (1) with the stratified distribution of parameters and initial conditions. For each state, we ran 100 simulations, each with initial conditions and parameters randomly drawn from the interquartile of the estimated distributions.

**1.2.1 Inference model**

To perform the inference in step 1) we used a county-scale metapopulation model in which transmission of SARS-CoV-2 is simulated within and between each of the 3142 counties of the US (*21*). The subpopulations of each county are linked by documented rates of inter-county commuting and random travel. The metapopulation model has a SEIR structure featuring the same distinction between reported and unreported cases, but without the age/exposure stratifications listed in Table S1. The metapopulation model is combined with the ensemble adjustment Kalman filter (*32*), which assimilates case observations (daily cases reported by The Center for System Science and Engeneering at John Hopkins University, JHU-CSSE) and iteratively estimates the time-evolving distribution of unobserved parameters and state variables. Details on the inference procedure can be found in (*21*). Using this framework, we obtained population level (i.e., unstratified) estimates of initial conditions on January 10, 2021 (e.g., the time-varying reproduction number *R_t_*, initial susceptibility, ascertainment rate, etc.).

**1.2.2 Mapping to stratified estimates**

We combined the inferred parameters with information on population structure, age specific seroprevalence estimates and published age-specific infection reporting rates as described in Supplementary Table S2.

**Table S2:** **Age- and population-specific parameter specifications.** All parameters and variables are state-specific; we omit the subscript _S=1:51_ for readability. *Estimated* parameters were inferred using the model-inference framework (*21*) (Section 1.2.1) and incidence data at the county level through January 10, 2021.

| PARAMETER | METAPOPULATION MODEL ESTIMATE | ADDITIONAL DATA | MODEL |
| --- | --- | --- | --- |
| Population structure N_i_ by state |  | - Census population structure for 50 states divided into the 5 age groups (*33*). - USAFACTS (*34*) for updated cumulative US population (N=3.2587e+08) - CDC ACIP PRIORITIZATION (*35,36*) | We divided the population in 12 groups (see Table S1).  We included all EW and HC in the two adult groups (18-49) and (50-64).  - 10% adults are HC, 30% adults are EW.  - 30% of the remaining adults (18-49) and (50-64) have risk factors for severe disease (RF).  - 39% of adults >65 have risk factors for severe disease (RF). |
| State Age-specific transmission rates $\boldsymbol{\beta}_{\boldsymbol{i,j}}$ | State specific R_0_ (non- stratified population), see Figure 1. | - *POLYMOD Study* (*37*) contact rates at home, school, work, others. - CDC modeling assumption: EW are able to reduce work contact 35% as much as the other groups can (*38*) | We define the transmission rate $\beta_{i,j}$so that the reproductive number matches the estimated R_0_ from Jan 10, 2021.  **Contact Matrix M= {**$\boldsymbol{c}_{\boldsymbol{i,j}}$**}:**  - Contact reductions to account for NPIs: 60% in school, 60% work, 30% others.  - EW work contact reductions: 20%  - HC work contact reductions: 0%  - Contacts of every group with adult group 18-45 and 45-64 are distributed within the 4 adult subgroups (HC, EW, RF, general) according to prevalence at *home* and *work;* and composed for 80% by EW and HC for *school* and *other* contacts.  Alternate estimates were tested in Supplementary Text S3.  **Transmission rate:**  $\beta_{i,j}=\beta_{0}c_{i,j}$where $\beta_{0}$is determined for each state by equating the R_t_ computed from the next generation matrix of system (1) to the R_t_ inferred with model in (*21),* following methods described in *(39).* |
| Ascertainment rates by age *i* and state *S*: $\boldsymbol{\alpha}_{\boldsymbol{i}}$ | $\alpha$non- stratified: median estimate $\alpha=0.23 ,$range across states: (0.19-0.35). | - Seroprevalence by age and State$p_{i}$ (latest data from end of September, 2020) *Nationwide Commercial Laboratory Seroprevalence Survey* (*40*) - Estimate $\alpha_{i,NY}$ of ascertainment rate by age group in NYC (estimate from December 15, 2020) (*41*) | We set the age-specific ascertainment rate proportional to Yang’s estimate from (*41*): $\alpha_{i}=k \alpha_{i,NY}$  where k verifies: $\alpha=\frac{\sum_{i} {\alpha_{i}p}_{i}N_{i}}{\sum_{i} p_{i}N_{i}}$  and $N_{i}$ and $p_{i}$are the population of age group i in state S and the seroprevalence in state S.  The same ascertainment rate is used for different categories of the same age group. |
| Ascertainment rates by age *i* and state *S* for vaccinated: $\tilde{\boldsymbol{a}_{\boldsymbol{i}}}$ |  |  | $\tilde{a_{i}}=\alpha_{i}$ in the baseline scenario. Alternate estimates considered in Supplementary Text S3. |
| Initial Susceptibility by age group *i* and state S: $\boldsymbol{S}_{\boldsymbol{0}_{\boldsymbol{i}}}$ | $S_{0}$ non- stratified, see Figure 1. | - Seroprevalence by age and State$p_{i}$ (latest data from November 2020) *Nationwide Commercial Laboratory Seroprevalence Survey* (*40*) | We set $S_{0_{i}}$ so that the sum over the groups is equal to the estimated $S_{0}$ on January 10, 2021 and the relative ratio of infection prevalence across age group matches the serological observations:  $S_{0_{i}}=N_{i}-u p_{i}N_{i}$  where u verifies  ${N-S}_{0}$=$u\sum p_{i}N_{i}$  and $N_{i}$ and $p_{i}$are the population of age group *i* in state S and the seroprevalence in state S.  The same seroprevalence estimate was used for different groups of same age.  Alternate estimates were tested in Supplementary Text S3. |
| Initial number of infected individuals by age and state:  $\boldsymbol{E}_{\boldsymbol{0}_{\boldsymbol{i}}}$**,** $\boldsymbol{I}_{\boldsymbol{0}_{\boldsymbol{i}}}$**,** $\boldsymbol{U}_{\boldsymbol{0}_{\boldsymbol{i}}}$ | $E_{0}$ **,** $I_{0}$ , $U_{0}$non- stratified | - Seroprevalence by age and State$p_{i}$ (latest data from November 2020)   *Nationwide Commercial Laboratory Seroprevalence Survey* (*40*) | We set initial condition for $E_{0_{i}}$, $I_{0_{i}}$, $U_{0_{i}}$ to match the seroprevalence ratio across age groups, the sum over the groups matches the posterior estimates for $E_{0}$ **,** $I_{0}$ , $U_{0}$on Jan. 10, 2021:  $E_{0_{i}}=h_{i} E_{0}$  where $h_{i}$=$\frac{p_{i}N_{i}}{\sum_{i} p_{i}N_{i}}$  and similarly, for $I_{0_{i}}$, $U_{0_{i}}$. |
| Duration of incubation **Z** | Z = 3.59 | Estimated using case data prior to March 13, 2020 in the US (*42*). | Assumed constant for all groups |
| Duration of infectious period **D** | D = 3.56 | Estimated using case data prior to March 13, 2020 in the US (*42*). | Assumed constant for all groups |
| Relative infectiousness ***r*** of unreported cases | *R* = 0.64 | Estimated using case data prior to March 13, 2020 in the US (*42*). | Assumed constant for all groups |
| R_0_ WITHOUT NPIs |  |  | 3 scenarios tested:  R_0_= (2.4; 2.8; 3.2) |
| CFR |  | CDC case report data Line List (*43*) | For the 12 groups of Table S1 the CFR is:  CFR(1) = 0.00044;  CFR(2) = 0.00099;  CFR(3:5) = 0.00139;  CFR(6) = 0.00367;  CFR(7:9) = 0.00409;  CFR(10) = 0.01739;  CFR(11) = 0.04463;  CFR(12) = 0.10979; |

**1.2.3 Vaccination-related parameters**

Vaccination administration in the US began on December 14, 2020. We assumed 5 million doses administered cumulatively in the first 3 weeks based on inoculation records (*8*). In subsequent weeks, beginning January 4, 2021, we assumed 5 million people vaccinated nationally for each subsequent week (except for the administration rate analysis in which weekly rates varied from 3 to 13 million vaccinated). We assumed both the BNT162b2 and mRNA-1273 vaccines required 2 doses and that complete efficacy was reached 1 week after the second dose. The time between first and second dose is 3 weeks for BNT162b2 and 4 weeks for mRNA-1273 (*4, 5*). Here, we averaged these two interval times and assumed that each individual received the second dose 3.5 weeks after the first dose. Weekly doses were assumed to be distributed uniformly over 7 days. Vaccine parameters and the administration timeline for the baseline scenario are detailed in Table S3. Vaccination in the simulations was administrated according to the following prioritization order based on (*3*):

1) Phase 1a: Healthcare workers (~20 million) & long-term care facility (LTCF) residents (~3 million).

2) Phase 1b: Front Line Essential Workers (~30 million) & adults >65 with RF (~14 million)

3) Phase 1c: Other Essential Workers (~30 million), adults >65 (~26 million) & adults with RF (37 million)

4) Other adults (~86 million)

5) Children (~79 million). Although both the BNT162b2 and mRNA-1273 vaccines are not currently recommended for children, in our analysis we hypothesize that by the end of the vaccination campaign vaccine will be recommended to all age groups.

In each of the 5 phases, individuals were immunized up to a target coverage (baseline target coverage was 80% for HC, 70% for adults ≥65 and adults with RF, and 60% for others.). Phases were considered completed for the purposes of NPI relaxation (used in some scenarios) 10 days after reaching target coverage (for first vaccination). The weekly timeline of baseline scenario vaccination is shown in Table S4.

**Table S3: Vaccination parameters.**

| **PARAMETER** | **SOURCE** | **ESTIMATE** |
| --- | --- | --- |
| VACCINE EFFICACY | Kaplan Meier curve for efficacy estimate (*4*) | $\epsilon_{1}$: 0% for 12 days after 1^st^ dose  $\epsilon_{2}:90\%$ from 12 days after 1^st^ dose to 7 days after 2^nd^ dose  $\epsilon_{3}:$95% from 7 days after 2^nd^ dose  Alternate estimate tested in Supplementary Text S3 |
| TIME BETWEEN DOSES | Pfizer- BNT162b2: 3 weeks (*4*)  Moderna-mRNA-1273: 4 weeks (*5*) | 3.5 weeks |
| TOTAL DOSES AVAILABLE | (*6, 7*) | 400 MILLION (enough to fully vaccinate 200 million people) |

**Table S4**: **Weekly (first) vaccine distribution timeline (in millions).** Weeks are identified by the first day of the week. Weekly doses are distributed uniformly over 7 days.

| WEEK | PEOPLE INITIATING VACCINATION | CUMULATIVE  POPULATION  INITIATING VACCINATION |
| --- | --- | --- |
| 14-Dec | 1 | 1 |
| 21-Dec | 1.5 | 2.5 |
| 28-Dec | 2.5 | 5 |
| 4-Jan | 5 | 10 |
| 11-Jan | 5 | 15 |
| 18-Jan | 5 | 20 |
| 25-Jan | 5 | 25 |
| 1-Feb | 5 | 30 |
| 8-Feb | 5 | 35 |
| 15-Feb | 5 | 40 |
| 22-Feb | 5 | 45 |
| 1-Mar | 5 | 50 |
| 8-Mar | 5 | 55 |
| 15-Mar | 5 | 60 |
| 22-Mar | 5 | 65 |
| 29-Mar | 5 | 70 |
| 5-Apr | 5 | 75 |
| 12-Apr | 5 | 80 |
| 19-Apr | 5 | 85 |
| 26-Apr | 5 | 90 |
| 3-May | 5 | 95 |
| 10-May | 5 | 100 |
| 17-May | 5 | 105 |
| 24-May | 5 | 110 |
| 31-May | 5 | 115 |
| 7-Jun | 5 | 120 |
| 14-Jun | 5 | 125 |
| 21-Jun | 5 | 130 |
| 28-Jun | 5 | 135 |
| 5-Jul | 5 | 140 |
| 12-Jul | 5 | 145 |
| 19-Jul | 5 | 150 |
| 26-Jul | 5 | 155 |
| 2-Aug | 5 | 160 |
| 9-Aug | 5 | 165 |
| 16-Aug | 5 | 170 |
| 23-Aug | 5 | 175 |
| 30-Aug | 5 | 180 |
| 6-Sep | 5 | 185 |
| 13-Sep | 5 | 190 |
| 20-Sep | 5 | 195 |
| 27-Sep | 5 | 200 |

**Supplementary Text S2: Extended model results on effect of NPIs.**

Figure S1 shows the cumulative attack rate and death rate beginning January 11, 2021 for the NPI scenarios described in Table S5 and 3 different estimates of R_0_ (R_0_=2.4; R_0_=2.8 and R_0_=3.2). In all scenarios involving vaccination, the timeline of vaccination followed the calendar in Table S4. Results in section *Effect of NPIs* in the main text correspond to Scenarios N0 to N5 of Table S5. Without interventions or vaccination (N0) the median national attack rate was 38% for R_0_=2.4, 45% for R_0_=2.8 and 50% for R_0_=3.2. Reductions due to NPIs were qualitatively consistent across the 3 estimates of R_0_, i.e. regardless of R_0_, the scenarios yielding the greatest reduction in infections and deaths nationally were N4, N5, N6 and N9. The best scenarios were therefore those in which NPIs were either first strengthened and then relaxed, or maintained at initial levels until 140 million people were vaccinated. Figure S2 compares the attack rate at the state level for the scenarios considered in the main text (N0 to N5). Among them, Scenario N4 and N5 had the lower attack rate for all states. At the national level, N4 averted more infections and deaths than N5, but in a few states where initial susceptibility was above 74% (Alaska, Vermont, Oregon, New Hampshire, Maine, Hawaii, Washington) N5 was substantially better than N4 (Figure S2).

**Table S5**: **All NPIs scenarios considered in the analysis**. Scenario N0 and N1 are, respectively, the non-intervention scenario and the only-vaccination scenario. In scenarios N2 to N6, relaxation of NPIs is triggered by milestones (phase completion or 140 million persons vaccinated). In Scenarios N7 to N9, relaxation of NPIs is triggered at specific time points.

| SCENARIO | VACCINATION | DESCRIPTION |
| --- | --- | --- |
| N0 | NO | NPIs immediately relaxed |
| N1 | YES | NPIs immediately relaxed |
| N2 | YES | NPIs maintained until completion of Phase 1a, then immediately relaxed |
| N3 | YES | NPIs maintained until completion of Phase 1a, then gradually relaxed upon completion of (1a,1b,1c) |
| N4 | YES | NPIs strengthened until completion of Phase 1a, then gradually relaxed upon completion of (1a,1b,1c) |
| N5 | YES | NPIs maintained until vaccination of 140 million people, then gradually relaxed in 3 1-month steps |
| N6 | YES | NPIs maintained until vaccination of 140 million people, then immediately relaxed |
| N7 | YES | NPIs as current, then immediately relaxed after 1 month |
| N8 | YES | NPIs as current, then gradually relaxed after 1 month in 5 1-months steps |
| N9 | YES | NPIs strengthened for 1 month then gradually relaxed.in 5 1-months steps |

**Figure S1 Attack rate and death rate for all NPIs.** Cumulative attack rate (first column), cumulative death rates (second column) and averted burden of infection and deaths (third column) for R0=2.4, R0=2.8, R0=3.2. Attack rate and death rate are measured as the fraction of the total population that was infected with SARS-COV-2 and the fraction of the total population that died between January 11 2021 through April 2022.

**Figure S2 Attack rate by state in scenarios N0 to N5.** Maps show the mean estimates of the attack rate (AR) for each state with R_0_=2.8 for the 6 scenarios N0 to N5.


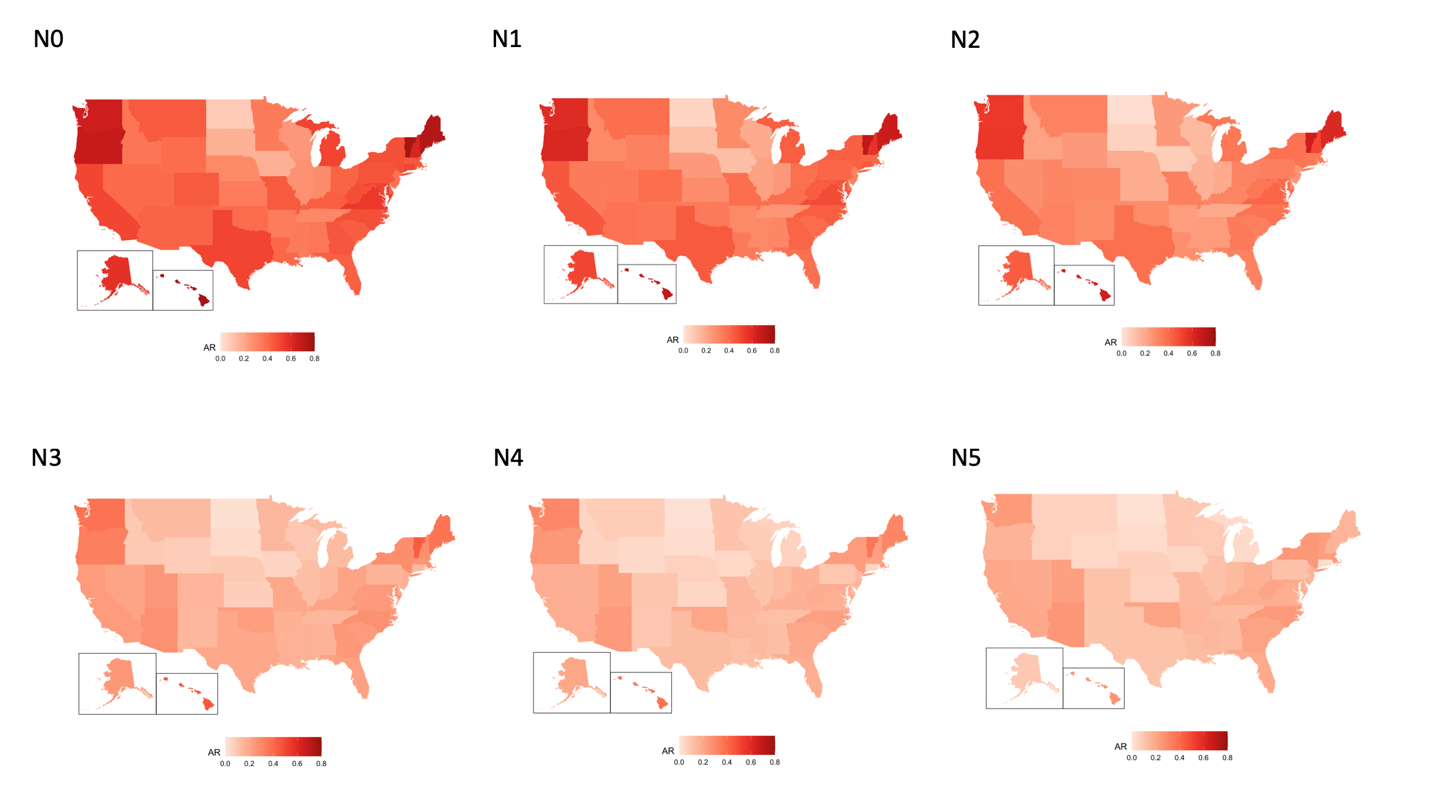


**Supplementary Text S3: Sensitivity Analysis**

**3.1 Sensitivity to variation of initial susceptibility**

The attack rate and death rate largely depended on the estimate of initial population susceptibility. Figure S3 shows the attack rate and death rate when initial susceptibility was increased or decreased 20% from the inferred estimate (65%), corresponding to 78% and 52% initial susceptibility, respectively. The median attack rate in scenario N0 varied from 27% in the decreased susceptibility sensitivity analysis to 62% in the higher susceptibility sensitivity analysis. The ranking of the 9 NPI scenarios of Supplementary Table 5 in terms of averted burden matched the original analysis, with scenarios N4, N5, N6 and N9 yielding the greatest reductions of infections and deaths. However, vaccination in the weaker NPI scenarios had a smaller relative impact when initial susceptibility was higher (Supplementary Figure S3).

**Figure S3**: **Sensitivity to variation of initial susceptibility.** Attack rate and death rate when initial susceptibility in each state is increased by 20% (upper panels) or decreased by 20% (lower panels). Here, R_0_=2.8, the vaccination calendar is shown in Table S4, and coverage is baseline c (80% HC, 70% for population >65 and adults with RF and 60% for others up to availability).

**3.2 Sensitivity to different estimates of vaccine efficacy**

The estimate of vaccine efficacy used in the model was based on results from the Pfizer and Moderna phase 3 trials (*4, 5*). However, experimental error (e.g. PCR sensitivity) and possible reduction of effectiveness due to new emerging viral variants (*27)* could impact the actual efficacy of the vaccine. When efficacy was reduced by 20% (both after the first and second dose), the averted infections and deaths were 70-95% and 75-98% of original analysis, respectively (Supplementary Figure S4).

**Figure S4:** **Attack rate and death rate for vaccine efficacy reduced by 20%**: ($\epsilon_{1}=0,$

$\epsilon_{2}=72\%$ $\epsilon_{3}=76\%$ ). Here R_0_=2.8, the vaccination calendar is presented in Table S4, and coverage is baseline c (80% HC, 70% for population > 65 and adults with RF and 60% for others up to availability).

**3.3 Sensitivity to variation of reduction in contact rates**

We tested the effect of varying the relative reduction of contact rates in matrix M of Supplementary Table S2 among population groups. The overall transmission rate was determined from the estimate of R_t_ for each state, derived from the metapopulation model, and by the NPIs scenario adopted; however, relative ratios of NPI-induced contact reduction to the POLYMOD contact rates (*37*) in different groups could be tuned. The values used in simulations were a 60% contact reduction in school, 60% contact reduction at work, no contact reduction at home and 30% contact reduction in other settings (Supplementary Table S2). Altering these percentages yielded less than 2% difference in attack rate but had a more substantial effect on deaths. However, the qualitative results were unchanged (Supplementary Figure S5).

**Figure S5 Sensitivity of the attack rate to varying the relative ratios of contact rates**. Results are for R_0_=2.8, the vaccine calendar in Table S4 and coverage baseline c (80% HC, 70% for population >65 and adults with RF and 60% for others up to availability). Symbols correspond to mean estimates of the attack rate and death rate corresponding to different choices of relative contact reduction due to NPIs. Red symbols are the mean estimate for the original setting (60% contact reduction in school, 60% contact reduction at work, no contact reduction at home, and 30% contact reduction in other settings). Black symbols are the mean estimates for 10 arbitrary perturbations of the relative contact reduction at school, work and other settings.

**3.4 Disease-blocking vaccine versus infection-blocking vaccine**

Vaccine trials demonstrated a 95% reduction of SARS-COV-2 disease in vaccine recipients. However, it is currently unclear whether the vaccine prevents infection or symptomatic disease upon infection. Throughout the analysis we assumed the former, by multiplying the transmission rate by the reduction constant $\varepsilon$. Here, we repeat the analysis assuming that vaccination does not prevent infection ($\varepsilon=1$), and assumes instead that the Exposed Vaccinated in system 1) are 95% less likely to develop a symptomatic infection ($\tilde{\alpha}=0.05*\alpha$) than the Exposed (unvaccinated). The first panel of Figure S6 shows the averted burden of the disease-blocking vaccine for R_0_=2.8 for all NPI scenarios in Table S5. Overall, averted infections were substantially reduced: averted infections in the disease-blocking vaccine were 1/3 of infection-blocking vaccine in some scenarios, whereas averted deaths were 85% to 95% of the infection-blocking vaccine scenarios.

**Fig S6. Comparison between attack rate and death rate for disease-blocking vaccination and infection-blocking vaccination.** Here, R_0_=2.8, vaccination calendar is as presented in Table S4 and coverage is baseline c (80% HC, 70% for population ≥65 and adults with RF and 60% for others up to availability).

**Supplementary Text S4: Extended model results for the effect of allocation rate**

Figure S7 extends the results of Figure 4 in the main text depicting the effect of allocation rate for 3 levels of R_0_. The qualitative behavior was similar across the different choices of R_0_: increasing the weekly deployment from 5 to 11 million doses yielded 7-14% more averted infections and 7-17% more averted deaths for R_0_=2.4; 9-16% more averted infections and 7-18% more averted deaths for R_0_=2.8; and 7-16% more averted infections and 8-20% more averted deaths for R_0_=3.2 with respect to scenario N0.

**Figure S7** **Averted burden for varying administration rates.** Panels show the fractional averted infections and deaths for the 4 time-triggered NPI scenarios N1(NO NPIs), N7 (LOW), N8 (MED), and N9 (HIGH) for 3, 5, 7, 9, 11 and 13 million individuals vaccinated each week, relative to the baseline scenario (N0). Each column displays the results corresponding to a different R_0_.

**Fig S8 Effect of NPIs and vaccination uptake on population immunity**. Panels A and B) Cumulative individuals no longer susceptible or infected (blue lines: recovered + deceased) and effectively vaccinated (red lines: recipients of vaccine who were susceptible) in scenario N1 (NO NPIs, panel A) and N9 (HIGH NPIs, panel B). Dashed blue and red lines refer to uptake scenario c and solid blue and red lines refer to uptake scenario c_0.5._ Black vertical dashed lines mark the end of vaccination (first doses) in c and c_0.5._  Panel C) Vaccine coverage of risk groups (adults with RF and population >65 with and without RF) through time from December 14, 2020 for the 6 uptake scenarios (c_0.5_, c_0.75_, c, c_1.2_, c_99_, c_R_).

**Supplementary Text S5 Extended model results on the effect of vaccine uptake**

Figure S9 extends the analysis of Figure 5 in the main text testing the effect of population uptake of vaccine for 3 levels of R_0_. The limited effect of uptake is consistent regardless of R_0._ Coverage scenario c_R_ yields the greatest reduction in deaths for all NPI scenarios and for all choices of R_0_. This moderate effect was not dependent on the cumulative number of doses: when we increased total available doses from 400 million to 600 million (from 200 million vaccinated to 300 million), and maintained an administration rate of 5 million vaccinations per week, the attack rate decreased by at most 0.003% in the stronger NPI scenarios and did not vary in the weaker NPI scenarios.

**Figure S9 Averted burden for varying uptake scenarios.** Panels show the fractional averted infections and deaths in the 4 time-triggered NPI scenarios N1 (NO NPIs), N7 (LOW), N8 (MED), N9(HIGH) for the 6 choices of population coverage (c_0.5_, c_0.75_, c, c_1.2_, c_99_, c_R_), 400 million total available doses, and 3 levels of R_0._ All numbers represent the fractional reduction relative to the baseline scenario (N0).

**Supplementary Text S6: Comparison with observed outcomes and interventions**

By July 4, 2021 183.26 million people aged 12 and older had received at least a first dose of vaccine in US (CDC), i.e. 55% of the total US population. The vaccination rate averaged 6.9 million vaccinations per week between January 10 and July 4 2021, but was not uniform: it increased from 5.6 million/week on the week of January 10 to 14.1 million/week on the week of April 4 and afterwards decreased to 1.6 million/week on the week beginning June 27 (*44*).

The average national time dependent reproductive number, R_t_, decreased for a short period after January 10 then increased from January 20 onward (Figure S10), qualitatively resembling NPI scenarios N4 and N9 (Figure S1, although the timing and magnitude of relaxation does not follow the prescribed values). It remains difficult to estimate the value of R_0_ in absence of restrictions, because:

1. R_0_ likely changed in recent months due to the emergence of new virus variants with higher transmissibility relative to the wild type (B.1.1.7. (alpha) circulating in US from February 2021 and B.1.617 (delta) from May 2021, (*45*));
2. The median estimate of R_t_ on July 4, 2021 in our model was 2.6. Even though many US states have almost completely relaxed all NPIs, there are still some residual measures in place like mask mandates and gathering restrictions, especially for children; and
3. It is likely that the current estimate of R_t_ reflects some seasonal forcing (sunlight, humidity, temperature) rendering the less virus transmissible than the first (winter) months of projections.

There were 11445041 reported cases from Jan 10 through July 4, 2021 in the US (*46*), accounting for 5.4% of the remaining susceptible population. Our estimate of the average reporting rate between January and July 2021, using prior methods (*21*) is 28%. Given these numbers, we can estimate a median attack rate of 19% of the population who were still susceptible on Jan 10, 2021. (We estimated 65% of the US population was still susceptible on January 10, 2021).

The resulting estimate of total infections is within the confidence intervals of scenarios N4 and N9 (Figure S1, although for these scenarios the vaccination rate was assumed stationary at 5 million doses/week) and scenario HIGH NPIs/7M in Figure S7. Also, note, the calculation assumes, erroneously, no repeat or vaccine breakthrough infections and thus, likely underestimates the population susceptibility.

There were 225199 reported deaths from Jan 10 through July 4 2021 (*46*), accounting for 0.0011% of the population estimated still susceptible on Jan 10 2021. Our model estimates for deaths in scenarios N4 and N9 (Figure S1 and S7) underestimate this total by a factor 2, possibly due to an underestimate of the case fatality rate (CFR). (note, the CFR was based on Line-List data (*43*) from late 2020 and with the following values for different age groups: CFR(0-4) = 0.00044; CFR(5-17) = 0.00099; CFR(18-49) = 0.00139; CFR(18-49 RF) = 0.00367; CFR(50-64) = 0.00409; CFR(50-64 RF) = 0.01739; CFR($\geq$65+) = 0.04463; CFR(65+ RF) = 0.10979.)

However, the case-fatality rate varies through time, and more recent estimates from 2021 suggest higher CFR values for 50-64 and 65+ both with and without risk factors.

Additionally, we only accounted for deaths corresponding to infections acquired after January 9, 2021. Given the large winter outbreak peaking in early January and the ~3-week delay from infection to reported death, we likely missed a substantial number of deaths caused by these earlier infections. In comparing what has played out in the last 6 months to the model results, note that the projection period in the model was 14 months, although herd immunity was reached well before the end in all scenarios.

**Figure S10**: Model-generated posterior estimates of national R_t_ from January 10 through July 4, 2021. Estimates were generated using the methods of (*21*).

**References**

(1) World Health Organization, Coronavirus disease (COVID-2019) situation reports, [cited 27 January 2021]. Available from:  [www.who.int/emergencies/diseases/novel-coronavirus-2019/situation-reports/](http://www.who.int/emergencies/diseases/novel-coronavirus-2019/situation-reports/)

(2) Covid 19 Vaccines, FDA [cited 4 January 2021]. Available from: <https://www.fda.gov/emergency-preparedness-and-response/coronavirus-disease-2019-covid-19/covid-19-vaccines>

(3**) Dooling K et al**. (2021) The advisory Committee on Immunization Practices’ Updated Interim Recommendation for Allocation of COVID-19 Vaccine – United States, December 2020. MMWR. Morbidity and Mortality Weekly Report; **69**: 1657:1660.

(4) **Polack FP et al**. (2020) Safety and Efficacy of the BNT162b2 mRNA Covid-19 Vaccine. New England Journal of Medicine; **383**: 2603:2615.

(5) **Baden LR et al**. (2021) Efficacy and Safety of the mRNA-1273 SARS-CoV-2 Vaccine. New England Journal of Medicine; **384**: 403:416.

(6) NYT, [cited 29 December 2020]. Available from: <https://www.nytimes.com/2020/12/23/us/politics/pfizer-vaccine-doses-virus.html>

(7) REUTERS, [cited 29 December 2020]. Available from: <https://www.reuters.com/article/us-health-coronavirus-moderna-idUSKBN28L2SY>

(8) CDC, Covid-19 Vaccination in the United States [cited 28 January 2021]. Available from: <https://covid.cdc.gov/covid-data-tracker/#vaccinations>

(9) REUTERS, [accessed 4 January 2021]. Available from: <https://www.reuters.com/article/us-health-coronavirus-usa-vaccinations-idUSKBN29512W>

(10) NYT, [cited 27 January 2021]. Available from: <https://www.nytimes.com/2020/12/02/health/Covid-Moderna-vaccine-children.html>

(11) REUTERS, [accessed 27 January 2021]. Available from: [https://www.reuters.com/article/brief-pfizer-says-covid-19-vaccine-trial idUSFWN2JX0V6](https://www.reuters.com/article/brief-pfizer-says-covid-19-vaccine-trial%20idUSFWN2JX0V6)

(12) **Li R et al**. (2020) Substantial undocumented infection facilitates the rapid dissemination of novel coronavirus (SARS-CoV-2), Science; **368**: 489:493.

(13) The Covid-19 Forecast Hub [cited 10 July 2021]. Available from: <https://covid19forecasthub.org>

(14) **Moore S et al.** (2021) Vaccination and non-pharmaceutical interventions for COVID-19: a mathematical modelling study. The Lancet Infectious Disease; **21**: 793-802.

(15) **Scientific Advisory Group for Emergencies**. (2021) Imperial College London: Unlocking roadmap scenarios for England, 18 February 2021; [accessed 10 July 2021]. Available from: <https://www.gov.uk/government/publications/imperial-college-london-unlocking-roadmap-scenarios-for-england-18-february-2021>.

(16) **Viana J et al.** (2021) Controlling the pandemic during the SARS-CoV-2 vaccination rollout. Nature Communication, **12**: 3674.

(17) **Giordano G et al.** (2021) Modeling vaccination rollouts, SARS-CoV-2 variants and the requirement for non-pharmaceutical interventions in Italy. Nature Medicine **27**: 993–998.

(18) **Betti M et al.** Integrated Vaccination and Non-Pharmaceutical Interventions based Strategies in Ontario, Canada, as a Case Study: a Mathematical Modeling Study. Journal of the Royal Society Interface. Published online 14 July 2021. doi: https://doi.org/10.1098/rsif.2021.0009.

(19) **Yang J et al.** (2021) Despite vaccination, China needs non-pharmaceutical interventions to prevent widespread outbreaks of COVID-19 in 2021. Nature Human Behaviour; **5**: 1009-1020.

(20) **Borchering R et al.** (2021) Modeling of Future COVID-19 Cases, Hospitalizations, and Deaths, by Vaccination Rates and Nonpharmaceutical Intervention Scenarios — United States, April–September 2021. MMMR. Morbidity and Mortality Weekly Report; **70**: 719–724.

(21) **Pei S, Kandula S, Shaman J**, (2020) Differential Effects of Intervention Timing on COVID-19 Spread in the United States. Science Advances, **6**: eabd6370.

(22) Midas Network, Covid-19 repository, [cited 28 January 2021]. Available from:

<https://github.com/midas-network/COVID-19/tree/master/parameter_estimates/2019_novel_coronavirus>

(23) CDC, Covid 19, Pandemic Planning Scenarios, [cited 13 February 2021]. Available from: <https://www.cdc.gov/coronavirus/2019-ncov/hcp/planning-scenarios.html>

(24) CNBC, [cited 27 January 2021]. Available from:

<https://www.cnbc.com/2021/01/26/biden-administration-orders-additional-200-million-doses-of-vaccine.html>

(25) **Bubar KM et al.** (2021) Model-informed COVID-19 vaccine prioritization strategies by age and serostatus. Science: **371**: 916-921.

(26) FDA Briefing Document, Moderna Covid-19 Vaccine, [cited 27 January 2021]. Available from: <https://www.fda.gov/media/144434/download>

(27) **Moore JP, Offit PA**. (2021) SARS-CoV-2 Vaccines and the Growing Threat of Viral Variants. JAMA - Journal of The American Medical Association, **325**: 821-822.

(28) BNO, Covid-19 reinfection tracker, [cited 27 January 2021]. Available from:

<https://bnonews.com/index.php/2020/08/covid-19-reinfection-tracker/>

# (29) Hall V et al. Do antibody positive healthcare workers have lower SARS-CoV-2 infection rates than antibody negative healthcare workers? Large multi-centre prospective cohort study (the SIREN study), England: June to November 2020. medRxiv [Preprint]. 2020 [cited 27 January 2021]. Available from: <https://www.medrxiv.org/content/10.1101/2021.01.13.21249642v1> (2021)

(30) **Letizia AG et al***.* (2021) SARS-CoV-2 seropositivity and subsequent infection risk in healthy young adults: a prospective cohort study. The Lancet Respiratory Medicine; **9**: 712-720.

(31) **Shaman J, Karspeck A.** (2012) Forecasting seasonal outbreaks of influenza. PNAS, 2012; **109**: 20425-20430.

(32) **Anderson L**, (2001) An ensemble adjustment Kalman filter for data assimilation. Monthly Weather Review; **129**, 2884–2903.

(33) US CENSUS [cited 10 December 2020]. Available from: <https://www.census.gov/programs-surveys/decennial-census/decade.html>

(34) USAFACTS [cited 10 December 2020]. Available from:

https://usafacts.org/data/

(35) ACIP December 20 meeting, CDC [cited 27 December 2020]. Available from: <https://www.cdc.gov/vaccines/acip/meetings/slides-2020-12.html>

(36) CDC*,* People at increased risk [cited 27 December 2020]. Available from: <https://www.cdc.gov/coronavirus/2019-ncov/need-extra-precautions/>

(37) **Prem K, Cook A, Jit M.** (2017) Projecting social contact matrices in 152 countries using contact surveys and demographic data. Plos Computational Biology; **13**: e1005697.

(38) CDC: Modeling strategies for the allocation of Sars-Cov-2 Vaccines. [cited 27 December 2020]. Available from: https:////www.cdc.gov/vaccines/acip/meetings/downloads/slides-2020-10/COVID-Biggerstaff.pdf

(39) **Diekmann O, Heesterbeek JA, Roberts MG**. (2010) The construction of next-generation matrices for compartmental epidemic models. Journal of the Royal Society Interface; **7**: 873-885.

# (40) CDC, Nationwide Commercial Laboratory Seroprevalence Survey, [cited 27 January 2021]. Available from: <https://www.cdc.gov/coronavirus/2019-ncov/cases-updates/commercial-lab-surveys.html>

(41) **Yang W et al.** (2021) Estimating the infection-fatality risk of SARS-CoV-2 in New York City during the spring 2020 pandemic wave: a model-based analysis. The Lancet Infectious Diseases; **21**: 203-212.

# (42) Pei S, Shaman J. (2020) Initial Simulation of SARS-CoV2 Spread and Intervention Effects in the Continental US. medRxiv [Preprint] 2020, [cited 7 January 2021]. Available from: https://www.medrxiv.org/content/10.1101/2020.03.21.20040303v2

(43) CDC, Line List [cited 10 January 2021]. Available from: [https://www.cdc.gov/epiinfo/user-guide/visual dashboard/analysisgadgets/linelist.html](https://www.cdc.gov/epiinfo/user-guide/visual%20dashboard/analysisgadgets/linelist.html)

(44) Vaccination Trends, COVID Data Tracker, CDC [cited 10 July 2021]. Available from <https://covid.cdc.gov/covid-data-tracker/#vaccination-trends>

(45) Variant Proportion, COVID Data Tracker, CDC [cited 10 July 2021]. Available from

<https://covid.cdc.gov/covid-data-tracker/?CDC_AA_refVal=https%3A%2F%2Fwww.cdc.gov%2Fcoronavirus%2F2019-ncov%2Fcases-updates%2Fvariant-proportions.html#variant-proportions>

(46) Covid-19 cases and deaths. [cited 10 July 2021]. Available from: https://ourworldindata.org
